# Reverse Shoulder Arthroplasty for the treatment of Proximal humeral fractures in the Elderly (ReShAPE trial) : study protocol for a multicentre combined randomised controlled and observational trial

**DOI:** 10.1186/s13063-017-1826-6

**Published:** 2017-02-28

**Authors:** Geoffrey C. S. Smith, Ed Bateman, Ben Cass, Maurizio Damiani, Wade Harper, Hugh Jones, David Lieu, Jeff Petchell, Minas Petrelis, Kalman Piper, Doron Sher, Christopher J. Smithers, John Trantalis, Sindy Vrancic, Ian A. Harris

**Affiliations:** 1 St. George and Sutherland Hospitals, Grey St, Kogarah, NSW Australia; 2 Gosford District Hospital, Holden St, Gosford, NSW Australia; 3 Royal North Shore Hospital, Reserve Rd, St. Leonards, NSW Australia; 4 The Canberra Hospital, 51 Jardine St, Kingston ACT, Australia; 5 Prince of Wales Hospital, Barker St, Randwick, NSW Australia; 6 Liverpool Hospital, Locked Bag 7103, Liverpool, NSW Australia; 7 Royal Prince Alfred Hospital, Missenden Rd, Camperdown, NSW Australia; 8 John Hunter Hospital, Lookout Rd, New Lambton Heights, NSW Australia; 9 Westmead Hospital, Hawkesbury Rd and Darcy Rd, Westmead, NSW Australia; 10 Concord Hospital, Hospital Road, Concord, NSW Australia; 11 Whitlam Orthopaedic Research Centre, Ingham Institute for Applied Medical Research, South Western Sydney Clinical School, UNSW Australia, Liverpool, NSW Australia; 12 Sydney Orthopaedic Trauma and Reconstructive Surgery, 5/19 Kensington St, Kogarah, Sydney, NSW 2217 Australia

**Keywords:** Fracture, Proximal humerus, Treatment, Surgery, Arthroplasty, Reverse shoulder arthroplasty

## Abstract

**Background:**

Proximal humeral fractures are common in older patients. The majority are minimally displaced and are associated with good outcomes after nonoperative treatment. Poorer outcomes are associated with displaced, multipart fractures. There is no clear benefit from surgical fracture fixation compared to nonoperative treatment. Replacement of the fractured humeral head with a hemiarthroplasty is another treatment option, but has not been shown to be clearly superior to nonoperative treatment or internal fixation. Recently, reverse total shoulder arthroplasty has been used to treat these fractures, particularly in the older population with several case series demonstrating good outcomes. No comparative trial has been performed to test the effectiveness of reverse total shoulder arthroplasty against nonoperative treatment.

**Methods/design:**

ReShAPE (Reverse Shoulder Arthroplasty for the treatment of Proximal humeral fractures in the Elderly) is a multicenter combined randomized and observational study. The primary objective is to compare pain and function 12 months post fracture using the American Shoulder and Elbow Society (ASES) score in patients aged 70 years or older with three- and four-part proximal humeral fractures treated by either reverse shoulder arthroplasty or nonoperative treatment. Secondary outcome measures will include the DASH (Disability of the Arm, Shoulder and Hand) score, the EQ-5D (EuroQol Health Survey), the EQ-VAS, pain, radiological parameters and complications.

**Discussion:**

The study will assess the effectiveness of reverse shoulder arthroplasty for complex proximal humeral fractures and thereby guide treatment of a common injury in the older population.

**Trial registration:**

World Health Organization Universal Trial Number (WHO UTN): U1111-1180-5452. Registered on 10 March 2016.

Australian and New Zealand Clinical Trials Registry (ANZCTR): 12616000345482. Registered on 16 March 2016.

**Electronic supplementary material:**

The online version of this article (doi:10.1186/s13063-017-1826-6) contains supplementary material, which is available to authorized users.

## Background

Humeral neck fractures account for 5% of fractures of the appendicular skeleton [1] and are the third commonest osteoporotic fracture [2] occurring with an incidence of 6.6 per 1000 person years [3]. There is a unipolar age distribution with most occurring in the older independent population with osteoporosis who fall from a standing height [4]. This incidence is set to increase in the next 20 years as a result of population growth and an aging population [5]. Forty-nine to 85% of proximal humeral fractures are minimally displaced and are usually treated nonoperatively with most having a good outcome regardless of comminution [4, 6, 7]. The poorer outcomes associated with displaced, multipart fractures has led surgeons to investigate operative alternatives [3, 8, 9]. Internal fixation with locking plates, which have some advantages in osteoporotic bone, have been subject to randomized trials, but have not been shown to improve outcome over nonoperative treatment [10–13]. Replacement of the fractured humeral head (hemiarthroplasty) is another treatment option, but has not been shown to be clearly superior to nonoperative treatment or plate fixation [14, 15].

Recently, reverse total shoulder arthroplasty has been used to treat these fractures, with several case series published [16–20]. This prosthetic design negates the effects of tuberosity malunion and nonunion that are common after internal fixation or hemiarthroplasty by creating a mechanical advantage for the deltoid muscle to allow active forward elevation and abduction. Studies comparing reverse total shoulder arthroplasty to hemiarthroplasty for the treatment of proximal humeral fractures have shown improved pain scores and functional outcomes after reverse shoulder arthroplasty [21, 22]. The use of reverse shoulder arthroplasty for the treatment of proximal humeral fractures is increasing [23]. Reverse shoulder arthroplasty has been reported to have a high complication rate including instability, loosening, poor rotation and radiological notching [24]. The risk of complications and prosthetic longevity limits the use of reverse shoulder arthroplasty in young patients with most prostheses being inserted in patients aged over 65 years [25].

## Methods/design

### Study objectives

We aim to test the hypothesis that in patients aged 70 years and over with three- or four-part fractures of the humeral neck, treatment with reverse total shoulder arthroplasty will result in improved shoulder pain and function 2 years post injury compared to nonoperative treatment.

### Study design

**Fig. 1:**
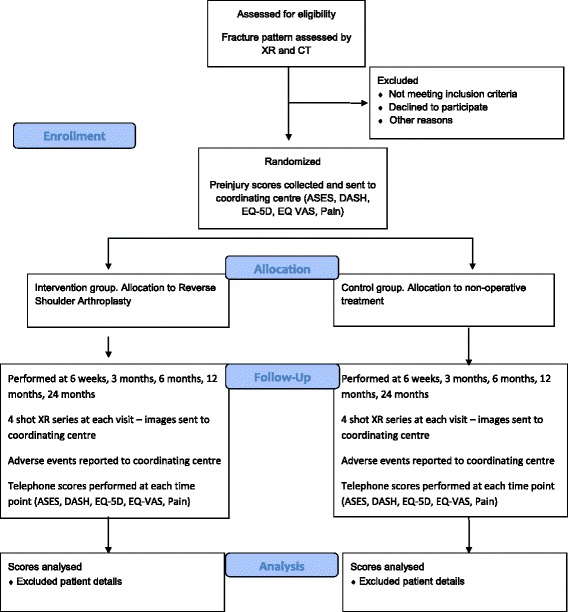
Consolidated Standards of Reporting Trials (CONSORT) flowchart

**Fig. 2:**
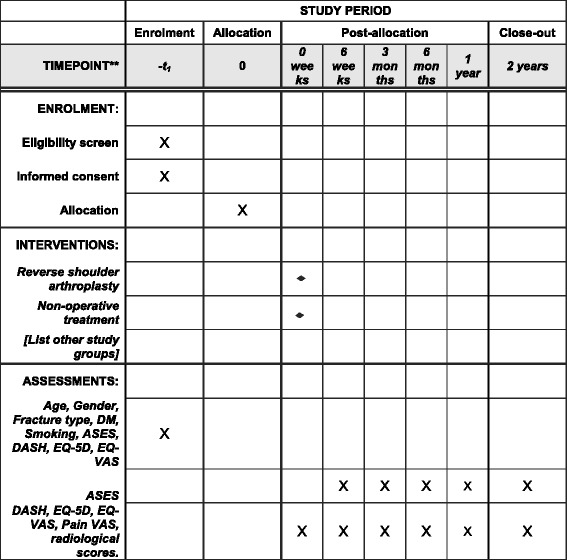
Standard Protocol Items: Recommendations for Interventional Trials (SPIRIT) diagram

The study is a combined multicenter randomized controlled trial (RCT) combined with an observational cohort study. Eleven centers in Australia will participate. The study Consolidated Standards of Reporting Trials (CONSORT) flowchart is provided in Fig. 1. The SPIRIT Checklist is provided in Additional file 1 and the Standard Protocol Items: Recommendations for Interventional Trials (SPIRIT) diagram is provided in Fig. 2

### Ethics

The recruiting sites in alphabetical order are: The Canberra Hospital, Concord Hospital, Gosford Hospital, John Hunter Hospital, Liverpool Hospital, Prince of Wales Hospital, Royal North Shore Hospital, Royal Prince Alfred Hospital, St. George Hospital, Sutherland Hospital and Westmead Hospital.

The study is registered with the Australasian and New Zealand Clinical Trials Registry (12616000345482) and the World Health Organization Universal Trail Registry (U1111-1180-5452)

### Study population

Patients will be considered for the study if they are 70 years of age or older, medically fit for surgery, have a three- or four-part proximal humerus fracture according to Neer’s classification [6] (Table 1), present within 28 days after injury, and are available for follow-up for 24 months.

**Table 1:** Neer’s classification of proximal humeral fractures and radiological inclusion criteria

Potential fracture “parts” (Neer)	Head (H), Shaft (S), Lesser tuberosity(LT), Greater tuberosity (GT)
Definition of a “part” (Neer)	>1 cm displacement or >45° rotation. Normal neck shaft angle 130°
Radiological inclusion criteria	3-part fractures: H + GT, S and LT H + LT, S and GT H, LT + GT and S H, GT, LT + S H, LT, GT + S 4-part fractures: H, LT, GT and S

Patients will be excluded if they are unable to provide consent due to limited proficiency in English or cognitive impairment (three or more errors on the Mini Mental Status Examination 6-item screening tool) [26], live in a residential aged care facility, have a pre-existing injury or condition of the affected side which severely affects the movement of the shoulder, a pathological fracture, other injury to the same upper limb requiring surgery, an open fracture, a fracture-dislocation or head-splitting fracture, a glenoid fracture, gross fracture displacement (no bony contact between humeral shaft and articular segment) or an axillary nerve palsy.

### Recruitment

Patients will be approached on presentation to a participating institution either in the emergency department or in the outpatient setting. Patients aged 70 years and over with a displaced proximal humerus fracture with tuberosity involvement will undergo a computed tomography (CT) scan with 3D reconstructions to assess the fracture configuration and to assess the number of fracture parts according to Neer’s classification [6] (Table 1). Linear measurements will be made with digital calipers. The normal neck shaft angle will be assumed to be 130°. Verbal consent will be requested to allow questioning to assess eligibility and to collect identifiable data. The fracture type will be screened against the inclusion criteria by two of the principal investigators. Patients who satisfy the study criteria will be invited to participate in the study and will receive a Participant Information Sheet (see Additional file 1). Patients who are willing to participate in the randomized trial will be asked to sign a Consent Form. Basic demographic and radiographic data will be collected for both groups to assess independent variables (age, gender, fracture type, diabetes (yes/no), smoking status (current/non), baseline shoulder function (American Shoulder and Elbow Society (ASES) score, Disability of the Arm, Shoulder and Hand (DASH) score) and baseline health (EuroQol Health Survey 5 dimensions (EQ-5D) and EuroQol Visual Analog Scale (EQ-VAS)). Patients who are unwilling to participate in the randomized trial will be offered participation in the observational study and will be provided with a separate Participant Information Sheet and Consent Form.

### Randomization

Randomization will occur immediately after consent has been gained. Randomization will occur by using a central, telephone-based automated randomization service with each site having its own access code (provided by the NHMRC Clinical Trials Center). Randomization will be stratified by site and will use minimization to adjust for gender and age (70–80 years, over 80 years).

### Interventions

Participants who are randomized to surgery will be treated by insertion of a reverse total shoulder arthroplasty within 28 days of the date of injury. Surgical technique (approach, version, component fixation and prosthesis choice) will be left to the discretion of the treating surgeon. The glenosphere will be placed low, avoiding superior tilt. The tuberosities will be repaired using nonabsorbable sutures. Postoperatively, the arm will be placed in a shoulder immobilizer and patients will be instructed on elbow, wrist and hand exercises to commence immediately. Two weeks postoperatively, pendular exercises and passive flexion to 90° and passive external rotation to neutral will be commenced. Unrestricted passive and active assisted exercises will be allowed, graduating to active mobilization (as tolerated) at 6 weeks post surgery. Resisted range of motion exercises will be allowed after 12 weeks. A minimum of five physiotherapy contacts within 3 months of treatment will be provided.

Patients randomized to nonoperative treatment will be placed in a shoulder immobilizer. Elbow wrist and hand exercises will be allowed immediately. After 2 weeks, pendular exercises and passive flexion to 90° and passive external rotation to neutral will be commenced. Unrestricted passive and active assisted exercises will be allowed graduating to active mobilization (as tolerated) at 6 weeks. Resisted range of motion exercises will be allowed after 12 weeks. A minimum of five physiotherapy contacts within 3 months of treatment will be provided.

### Parallel observational cohort study

Patients who do not consent to be randomized will be offered participation in the observational arm of the study. Their treatment will consist of the same two treatment options as the RCT arm. Treatment will be decided by patient and surgeon preference as per usual practice at each institution. Treatment protocols, follow-up and outcome measures will be the same as the randomized arm. The outcome of this arm of the trial will be analyzed separately. The inclusion of the observational cohort will inform the generalizability of the randomized trial.

### Outcome measures

Participants will be followed up at 6 weeks, 3 months, 6 months, 1 year, 2 years, 5 years and 10 years. The outcome measures listed below will be assessed at 6 weeks, 3 months, 6 months, 1 year, 2 years, 5 years and 10 years.

The primary outcome will be the ASES (American Shoulder and Elbow Society) standardized shoulder assessment score patient self-reported section at 24 months [27]. This scoring system consists of two dimensions: pain and activities of daily living, which are both equally weighted giving a total score out of 100, with higher scores indicating less pain and better function. It has been shown to be reliable, valid and responsive in shoulder arthroplasty and fracture [28, 29]. Secondary outcome measures will include DASH (Disability of the Arm, Shoulder and Hand) score [30], the EQ-5D and the EQ-VAS, pain (verbal numerical rating scale, 0–10 points), radiological parameters and complications (repeat shoulder surgery, readmission, infection requiring treatment, neurological deficit, dislocation, death). Radiographs will be performed at each follow-up and will include a four-radiograph series: an AP view in neutral and 30° external rotation, and a transthoracic scapula “Y” view and axillary view. Radiographic parameters that will be assessed are: healing of the tuberosities (nonunion or resorption will be considered failures to heal), position of the tuberosities, scapula notching (according to the Sirveaux classification system [31]) and loosening and alignment (coronal and sagittal plane for patients treated nonoperatively). All radiographs will be assessed by two independent observers independently. In case of discrepancy the scoring will be decided by discussion.

#### Sample size calculation

Case series documenting the outcome after reverse shoulder arthroplasty have shown a standard deviation (SD) of 13 in the ASES score. The minimum important clinical difference (MICD) in ASES scores has been estimated to be 6.4–18 [28, 32]. We consider a difference of 10 to be necessary in order to justify the additional costs and risks of surgery. A total of 60 patients (30 in each group) will provide 80% power to detect a MICD of 10 points at a significance of 0.05. We aim to recruit 72 patients to allow for 17% loss to follow-up. The ASES score after reverse shoulder arthroplasty has been reported to be 68–86 (higher score being better) [33]. In a paper reporting the outcomes of two-, three- and four-part fractures (our study proposal is limited to three- and four-part fractures) treated nonsurgically the mean ASES score was 82.5 [13].

#### Statistical analysis

Differences in baseline characteristics (i.e., intrinsic and injury-related variables) between both intervention groups will be assessed using the Student’ s *t* test (parametric continuous data), the Mann-Whitney *U* test (nonparametric continuous data) or the chi-square test (categorical data).

Unadjusted analysis by intention-to-treat will be performed to test the difference in primary and secondary outcomes between the intervention groups. Student’ s *t* test (parametric continuous data), the Mann-Whitney *U* test (nonparametric continuous data) or the chi-square analysis (categorical data) will be used. A *p* value < 0.05 (two-sided tests) will be taken as threshold of statistical significance.

The observational arm will be analyzed separately, comparing the same treatment groups against the same outcomes using multivariable linear regression to adjust for potential confounders (age, sex, handedness, smoking, fracture type).

### Monitoring and quality assurance

Adverse events to be recorded are repeat shoulder surgery, readmission, infection requiring treatment, neurological deficit, dislocation and death. All adverse outcomes will be recorded centrally.

Data will be collected by local site investigators and study documents will be submitted securely (scanned and emailed) to the project manager at the administering institution. Identifying data will be transferred separately from any study information and stored separately to the study database. All research documentation will be labelled with a unique person number as an identifier and stripped of any potentially identifiable information. Data will be stored in password-protected computers and locked filing cabinets as required within the administering institution

### Blinding

Study investigators, surgeons and participants will not be blinded to the treatment group due to the nature of the comparators (surgery versus no surgery). The primary outcome (ASES score) and secondary outcome scores will be collected by blinded researchers by telephone at all time points. The statistician will be blinded to the treatment group

## Discussion

The optimal treatment of three- and four-part fractures of the proximal humerus in older people has not yet been established. Operative treatment in the form of internal fixation or hemiarthroplasty has not been shown to be beneficial over nonoperative treatment. The design of the reverse shoulder arthroplasty has theoretical advantages in the treatment of these difficult fractures and several case series have shown good outcomes after reverse shoulder arthroplasty. However, there are no prospective RCTs comparing reverse shoulder arthroplasty to nonoperative treatment of displaced proximal humeral fractures in older people.

The ReShAPE trial will compare these treatment modalities and will guide future treatment for this common injury, potentially changing current practice.

The use of multiple different centers, variability in selection of surgical technique (approach, version, component fixation and prosthesis choice) and an observational cohort will increase the generalizability of the results. Randomization will occur only after review of the scans and after eligibility has been determined; therefore, making the two groups comparable and reducing selection bias.

### Trial status

The ReShAPE trial commenced recruitment in March 2016 but this has not yet been completed.

## Additional file

Additional file 1: SPIRIT 2013 Checklist: recommended items to address in a clinical trial protocol and related documents*. (DOC 121 kb)

## Abbreviations

ASES: American Shoulder and Elbow Society
DASH: Disability of the Arm, Shoulder and Hand
